# Identifikation gelenkspezifischer Risikofaktoren für die Risikoadjustierung in der Knieendoprothetik – ein modifiziertes Delphi-Verfahren

**DOI:** 10.1007/s00132-026-04768-2

**Published:** 2026-02-17

**Authors:** Dirk Müller, Holger Haas, Hartmut Bork, Johannes Flechtenmacher, Andreas Halder, Karl-Dieter Heller, Bernd Kladny, Jörg Lützner, Wolfram Mittelmeier, Carsten Perka, Andreas Pingsmann, Maximilian Rudert, Arnd Steinbrück, Dieter Christian Wirtz, Amelie Flothow, Leonie Sundmacher, Igor Lazic, Rüdiger von Eisenhart-Rothe

**Affiliations:** 1 Klinik und Poliklinik für Orthopädie und Sportorthopädie, TUM Klinikum Rechts der Isar, Ismaninger Str. 22, 81675 München, Deutschland; 2 Allgemeine Orthopädie, Unfallchirurgie und Sportmedizin, Gemeinschaftskrankenhaus Bonn, Haus St. Petrus, Bonner Talweg 4–6, 53113 Bonn, Deutschland; 3 Rehazentrum am St. Josef Stift, Westtor 7, 48324 Sendenhorst, Deutschland; 4 Ortho-Zentrum, Orthopädische Gemeinschaftspraxis am Ludwigsplatz, Waldstraße 67, 76133 Karlsruhe, Deutschland; 5 Klinik für Operative Orthopädie, Sana Kliniken Sommerfeld, Waldhausstraße 44, 16766 Kremmen, Deutschland; 6 Orthopädische Klinik, Stiftung Herzogin Elisabeth Hospital, Leipziger Straße 24, 38124 Braunschweig, Deutschland; 7 Abteilung für Orthopädie und Unfallchirurgie, m&i-Fachklinik Herzogenaurach, In der Reuth 1, 91074 Herzogenaurach, Deutschland; 8 UniversitätsCentrum für Orthopädie, Unfall- & Plastische Chirurgie, Universitätsklinikum Carl Gustav Carus Dresden, Fetscherstraße 74, 01307 Dresden, Deutschland; 9 Orthopädische Klinik und Poliklinik, Universitätsmedizin Rostock, Schillingallee 35, 18057 Rostock, Deutschland; 10 Centrum für Muskuloskeletale Chirurgie, Charité – Universitätsmedizin Berlin, Charitéplatz 1, 10117 Berlin, Deutschland; 11 Orthopädische Gemeinschaftspraxis in der Biberburg, Gatower Str. 241–243, 14089 Berlin, Deutschland; 12 Orthopädische Klinik König-Ludwig-Haus, Universität Würzburg, Brettreichstraße 11, 97074 Würzburg, Deutschland; 13 OCKA orthopädisch chirurgisches Kompetenzzentrum Augsburg, Vinzenz-von-Paul-Platz 1, 86152 Augsburg, Deutschland; 14 Klinik und Poliklinik für Orthopädie und Unfallchirurgie, Universitätsklinikum Bonn, Sigmund-Freud-Straße 25, 53127 Bonn, Deutschland; 15 Fachgebiet für Gesundheitsökonomie, Technische Universität München, TUM Campus im Olympiapark, Am Olympiacampus 11, 80809 München, Deutschland

**Keywords:** Delphi-Methode, Expertenkonsens, Gelenkspezifische Risikofaktoren, Risikoadjustierung, Totaler Kniegelenkersatz, Delphi method, Expert consensus, Joint-specific risk factors, Risk adjustment, Total knee replacement

## Abstract

**Hintergrund:**

Eine valide Risikoadjustierung ist in der Endoprothetik Voraussetzung, um die Behandlungsqualität zwischen Kliniken und Operateuren vergleichbar zu machen. Bestehende Modelle berücksichtigen überwiegend patientenspezifische Faktoren wie Alter oder Komorbiditäten. Hinsichtlich gelenkspezifischer Risikofaktoren für postoperative Komplikationen besteht bisher kein Konsens.

**Fragestellung:**

Ziel dieser Studie war die Erarbeitung eines Expertenkonsensus zu klinisch relevanten gelenkspezifischen Risikofaktoren in der Knieendoprothetik.

**Material und Methode:**

Ein dreistufiges modifiziertes Delphi-Verfahren wurde mit 14 deutschen Experten, die in Diagnostik und Therapie in der Knieendoprothetik eingebunden sind, durchgeführt. Auf Basis einer Literaturrecherche wurden 120 potenzielle Risikofaktoren identifiziert. In Runde 1 erfolgte deren Bewertung, in Runde 2 eine Fokussierung auf 70 gelenkspezifische Faktoren. In einer virtuellen Konsenssitzung (Runde 3) wurden die wichtigsten Faktoren verfeinert und priorisiert.

**Ergebnisse:**

Es konnten 12 gelenkspezifische Risikofaktoren identifiziert und nach Relevanz eingeordnet werden: septische Voroperationen, große Knochendefekte, einliegendes Fremdmaterial, Valgusfehlstellung > 10°, Varusfehlstellung > 15°, Streckdefizit > 10°, Flexion < 70°, Kellgren-Lawrence-Score < 3°, Patella baja, knöcherne Voroperation, Genu recurvatum > 10° sowie vorausgegangene Bandrekonstruktionen.

**Schlussfolgerungen:**

Im Rahmen eines strukturierten Delphi-Konsensusverfahrens konnten erstmals 12 gelenkspezifische Risikofaktoren durch Experten im deutschsprachigen Raum definiert werden. Diese Risikofaktoren können bestehende Risikoadjustierungsmodelle ergänzen. Zukünftige Studien sollten ihre prognostische Validität prüfen und die Integration in Routinedatenregister (z. B. EPRD) sowie Qualitätssicherungsinstrumente evaluieren.

**Graphic abstract:**

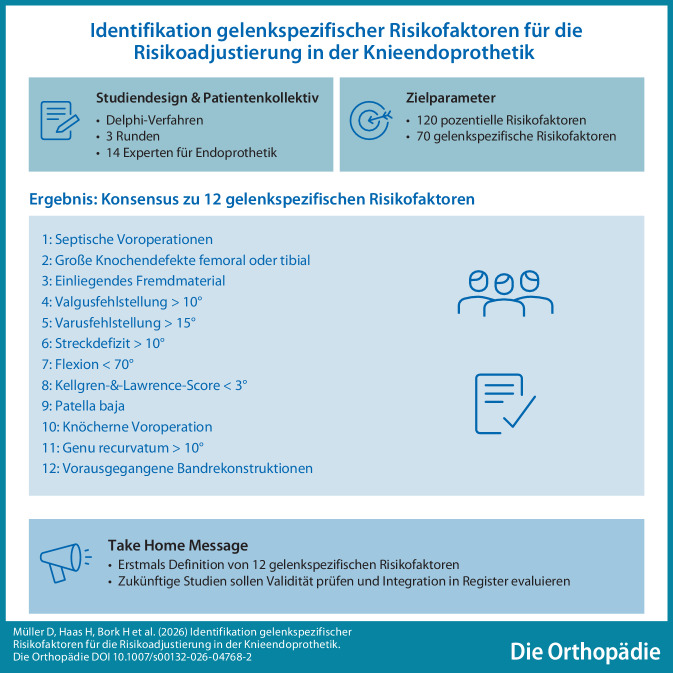

## Abstract (en)

**Background:**

Valid risk adjustment is a prerequisite in knee arthroplasty to enable fair comparisons of treatment quality across hospitals and surgeons. Existing models mainly consider patient-related factors such as age or comorbidities. Consensus on joint-specific risk factors for postoperative complications is currently lacking.

**Objectives:**

The aim of this study was to establish expert consensus on clinically relevant joint-specific risk factors in total knee arthroplasty (TKA).

**Methods:**

A three-stage modified Delphi process was conducted with 14 German experts in the field of TKA. Based on a systematic literature search, 120 potential risk factors were identified. In the first round, all factors were rated; in the second round, the focus was narrowed to 70 joint-specific factors. In a final virtual consensus meeting (third round), the most relevant factors were refined and prioritized.

**Results:**

Twelve joint-specific risk factors were identified and ranked: prior septic surgery, large bone defects, implanted foreign material, valgus malalignment > 10°, varus malalignment > 15°, extension deficit > 10°, flexion < 70°, Kellgren–Lawrence score < 3°, patella baja, prior bone surgery, genu recurvatum > 10°, and prior ligament reconstruction.

**Conclusions:**

Through a structured Delphi consensus process, 12 joint-specific risk factors were defined by a panel of German arthroplasty experts. These factors may meaningfully complement existing risk adjustment models. Future research should evaluate their predictive validity and integration into routine registries (e.g., EPRD) as well as quality assurance instruments.

## Hintergrund

Die Implantation einer Kniegelenksendoprothese (Knie-TEP) gehört zu den häufigsten und erfolgreichsten Verfahren zur Behandlung der fortgeschrittenen Gonarthrose. Sie ermöglicht eine deutliche Schmerzreduktion und Wiederherstellung der Gelenkfunktion. Dennoch treten postoperative Komplikationen – von Wundheilungsstörungen über Infektionen bis hin zum Implantatversagen – in 1–6 % der Fälle auf [4, 5, 25, 33]. Ihr Auftreten hängt wesentlich von patientenspezifischen, gelenkspezifischen und prozedurspezifischen Faktoren ab [4, 16]. Die frühzeitige Identifizierung von Risikopatientinnen und -patienten ist daher entscheidend, um Behandlungsergebnisse zu optimieren und das perioperative Management gezielt anzupassen.

Zusätzliche Relevanz erhält das Thema durch aktuelle gesundheitspolitische Entwicklungen: Mit Inkrafttreten des Krankenhaus-Transparenzgesetzes (19. Oktober 2023) [8] und der Einführung des Bundes-Klinik-Atlas (1. Mai 2024) [7] wird eine transparente und vergleichbare Darstellung von Behandlungsergebnissen gefordert. Voraussetzung dafür sind präzise Risikoadjustierungsmodelle, die eine faire Bewertung der Versorgungsqualität zwischen Kliniken ermöglichen.

Bisherige Modelle berücksichtigen überwiegend patientenbezogene Faktoren wie Alter, Geschlecht, Body-Mass-Index (BMI) und Komorbiditäten, darunter Diabetes mellitus, kardiovaskuläre Erkrankungen oder Nikotinkonsum [3–5, 11, 14, 27–30, 33]. Häufig werden diese mithilfe des Elixhauser-Scores [15] erfasst, wie etwa in den Risikoadjustierungen des Wissenschaftlichen Instituts der AOK (WidO) [34]. Für die Mortalitätsbewertung nach Knie-TEP durch das IQTIG wurden 2024 die Faktoren Geschlecht, Alter, Gehstrecke bei Aufnahme, Verwendung von Gehhilfen, ASA-Klassifikation, präoperative Wundkontamination, periprothetische Fraktur als Indikator und Implantation einer unikondylären Schlittenprothese berücksichtigt [22].

Obwohl diese Modelle wertvolle Erkenntnisse liefern, sind sie für die individuelle Risikoprognose häufig zu unspezifisch [4, 16]. Prozedurspezifische Parameter in der Knieendoprothetik – wie Kopplungsgrad der Prothese oder Zertifizierung des Leistungsträgers – werden bislang nicht allumfassend systematisch erfasst, obwohl sie aus klinischer Sicht potenziell entscheidende Zusatzinformationen bieten könnten. Auch gelenkspezifische Parameter – wie Gelenksdeformitäten, Bewegungsumfang, Achsverhältnisse oder strukturelle Anomalien – die im klinischen Kontext bereits erfasst werden, sind bisher nur unzureichend berücksichtigt.

Ein einheitlicher wissenschaftlicher Konsens zu klinisch relevanten gelenkspezifischen Risikofaktoren fehlt bislang. Diese Forschungslücke erschwert die Entwicklung valider und umfassender Risikomodelle erheblich. Ziel der vorliegenden Studie war es daher, im Rahmen eines strukturierten Delphi-Verfahrens mit einem interdisziplinären Expertenpanel einen Konsens zu relevanten gelenkspezifischen Risikofaktoren in der Knieendoprothetik zu erarbeiten. Auf dieser Grundlage soll eine verbesserte Risikoadjustierung ermöglicht werden, die künftig präzisere Qualitätssicherung und bessere Vergleichbarkeit in der endoprothetischen Versorgung unterstützt.

## Material und Methode

### Delphi-Verfahren

Wir führten ein dreistufiges Delphi-Verfahren durch, das sich methodisch an der RAND/UCLA Appropriateness Method (RAM) orientierte. Diese Vorgehensweise verbindet systematisch den aktuellen wissenschaftlichen Erkenntnisstand mit klinischer Expertise und bietet damit einen strukturierteren Rahmen als klassische Delphi-Leitlinienverfahren. Das hier vorgestellte Verfahren wurde dankenswerterweise durch die Arbeitsgemeinschaft der Wissenschaftlichen Medizinischen Fachgesellschaften e. V. (AWMF) begleitet und unterstützt. Durch die Kombination einer systematischen Literaturrecherche mit panelbasierten Bewertungen wurde sichergestellt, dass die identifizierten Risikofaktoren sowohl evidenzbasiert als auch praxisrelevant sind [18]. Der Delphi-Prozess wurde im Zeitraum von 03.08.2023 bis 13.02.2024 durchgeführt und umfasste eine systematische Literaturrecherche, zwei Online-Befragungen (Runden 1 und 2) sowie eine abschließende virtuelle Panelsitzung zur finalen Konsensbildung (Runde 3).

### Zusammenstellung des Delphi-Panels

Die Rekrutierung des Delphi-Panels erfolgte deutschlandweit mit dem Ziel, eine interdisziplinäre und ausgewogen zusammengesetzte Expertengruppe zu bilden, die den gesamten prä-, intra- und postoperativen Behandlungsverlauf abbildet. Daher wurden Fachpersonen aus konservativer und operativer Orthopädie sowie aus stationären und ambulanten Versorgungseinrichtungen einbezogen. Die Panelgröße lag im empfohlenen Bereich von 7–15 Mitgliedern, was sowohl Vielfalt als auch eine aktive Beteiligung aller Teilnehmenden ermöglichte [18]. Zur Sicherstellung der fachlichen Expertise wurden ausschließlich erfahrene Orthopäden mit langjähriger klinischer Tätigkeit ausgewählt.

### Literaturrecherche

Vor Beginn des Delphi-Verfahrens erfolgte eine systemische Literaturrecherche in den Datenbanken MEDLINE (PubMed), Embase, Cochrane CENTRAL, ClinicalTrials.gov und Google Scholar um alle relevanten Risikofaktoren für Komplikationen nach Implantation von Knie-TEP zu identifizieren.

### Online-Fragebogen

Für die erste und zweite Delphi-Runde wurde ein elektronischer Fragebogen mit der Software SurveyMonkey erstellt. In der Einleitung erhielten die Panelmitglieder Informationen zu Zielsetzung und Ablauf der jeweiligen Runde, zu den Bewertungskriterien der Risikofaktoren sowie zu den Vorgaben zur Wahrung von Anonymität und Vertraulichkeit [13, 19]. Neben den Skalenbewertungen enthielt der Fragebogen ein Freitextfeld, in dem die Teilnehmenden Änderungen an bestehenden Risikofaktoren vorschlagen oder neue Faktoren ergänzen konnten.

Alle Panelmitglieder erhielten einen personalisierten Direktlink zum Fragebogen per E‑Mail. Während des gesamten Prozesses bestand zudem die Möglichkeit, das Forschungsteam für Rückfragen zu kontaktieren, um ein einheitliches Verständnis der Zielsetzungen und Abläufe sicherzustellen.

### Ablauf des Delphi-Verfahrens

In der ersten Delphi-Runde erhielten die Panelmitglieder detaillierte Informationen zu den Forschungszielen, zum Zweck des Verfahrens sowie zum methodischen Vorgehen. Anschließend bewerteten sie die aus der systematischen Literaturrecherche abgeleiteten Risikofaktoren auf einer Likert-Skala von −2 bis +2. Faktoren mit einem Mittelwert ≥ +1 wurden in die nächste Runde übernommen. Zusätzlich konnten die Teilnehmenden weitere Risikofaktoren vorschlagen, die ebenfalls in die zweite Runde einbezogen wurden. Da in der ersten Runde ein Konsens darüber bestand, dass die Gesamtzahl der Faktoren zu hoch war, erfolgte in der zweiten Runde eine gezielte Fokussierung auf gelenkspezifische Risikofaktoren. In der zweiten Runde bewerteten die Panelmitglieder die verbleibenden Faktoren mit „+“ oder „−“. Zusätzlich wurde eine Rangfolge der positiv bewerteten Risikofaktoren erstellt. Die dritte und abschließende Runde fand als virtuelle Sitzung über die Videokonferenzplattform Zoom statt [18]. Hier wurden die Ergebnisse präsentiert und die Risikofaktoren klar definiert. Abschließend einigte sich das Panel in der dritten Delphi-Runde konsensbasiert und einstimmig, ohne verbleibende Gegenstimmen, auf eine Liste der 12 wichtigsten gelenkspezifischen Risikofaktoren, die nach Relevanz priorisiert wurden.

Da im Rahmen der Delphi-Befragung ausschließlich fachliche Experteneinschätzungen erhoben und keine personenbezogenen oder patientenbezogenen Daten verarbeitet wurden, war kein formales Ethikvotum erforderlich. Die Teilnahme war freiwillig; alle Panelmitglieder wurden vor Beginn des Verfahrens über Ziele, Ablauf und Inhalte informiert und gaben ihr Einverständnis zur Teilnahme.

## Ergebnisse

### Zusammensetzung der Expertengruppe

Das finale Delphi-Panel setzte sich aus 14 deutschen Experten der Knieendoprothetik zusammen (Tab. 1). Die Studie wurde in Zusammenarbeit und mit Unterstützung der Deutschen Gesellschaft für Orthopädie und Orthopädische Chirurgie (DGOOC) durchgeführt.

**Tab. 1:** Expertenpanel der 14 Experten des Delphi-Verfahrens (Reihenfolge alphabethisch).

Name (Alphabetisch)	Klinik	Tätigkeit
Dr. Hartmut Bork	Reha-Zentrum am St. Josef-Stift Sendenhorst	Rehabilitation, Klinik
Prof. Dr. Rüdiger von Eisenhart-Rothe	Universitätsklinikum rechts der Isar, TU München	Operativ, Universitätsklinik
Dr. Johannes Flechtenmacher	Ortho-Zentrum am Ludwigsplatz, Karlsruhe	Konservativ, Niedergelassen
Dr. Holger Haas	Gemeinschaftskrankenhaus Bonn	Operativ, Maximalversorger
Prof. Dr. Andreas Halder	Sana Kliniken Sommerfeld	Operativ, Maximalversorger
Prof. Dr. Karl-Dieter Heller	Herzogin Elisabeth Hospital Braunschweig	Operativ, Maximalversorger
Prof. Dr. Bernd Kladny	Fachklinik Herzogenaurach	Rehabilitation, Klinik
Prof. Dr. Jörg Lützner	Universitätsklinikum Carl Gustav Carus Dresden	Operativ, Universitätsklinik
Prof. Dr. Wolfram Mittelmeier	Universitätsklinikum Rostock	Operativ, Universitätsklinik
Prof. Dr. Carsten Perka	Universitätsklinikum Charité Berlin	Operativ, Universitätsklinik
Dr. Andreas Pingsmann	Gemeinschaftspraxis Biberburg, Berlin	Operativ, Niedergelassen
Prof. Dr. Maximilian Rudert	König-Ludwig-Haus Würzburg, Universität Würzburg	Operativ, Universitätsklinik
Prof. Dr. Arnd Steinbrück	Orthopädisch chirurgisches Kompetenzzentrum Augsburg	Operativ, Niedergelassen
Prof. Dr. Dieter Wirtz	Universitätsklinikum Bonn	Operativ, Universitätsklinik

### Ergebnis der Literaturrecherche

**Abb. 1:**
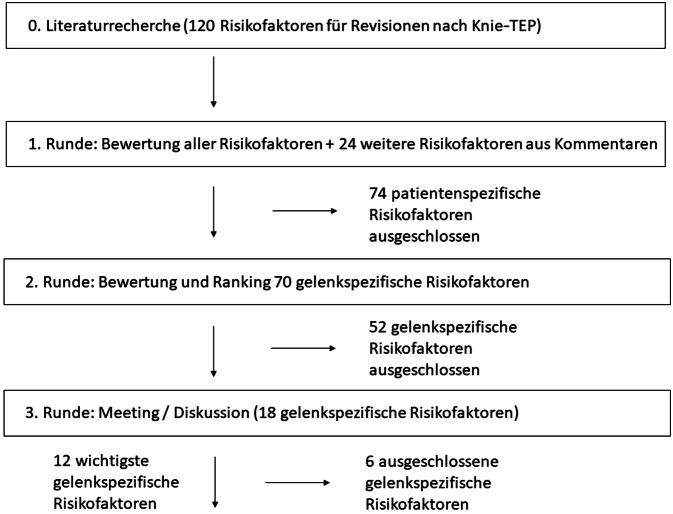
Ablauf des Delphi-Verfahrens; *TEP* Totalendoprothese

Im Rahmen der systematischen Literaturrecherche konnten 120 potenzielle Risikofaktoren für Komplikationen nach Implantation einer Knie-TEP identifiziert werden, diese wurden in die erste Runde des Delphi-Verfahrens übernommen. Der Ablauf des Delphi-Verfahrens ist in Abb. 1 schematisch zusammengefasst.

### Ergebnisse der 3 Runden des Delphi-Verfahrens

In der ersten Runde bewerteten die Panelmitglieder die 120 aus der Literaturrecherche abgeleiteten Risikofaktoren. Ergänzend wurden, auf Grundlage der Kommentare der Teilnehmenden, 24 weitere Faktoren aufgenommen. Da Einigkeit darüber bestand, dass die Gesamtzahl zu hoch war, konzentrierte sich die zweite Runde ausschließlich auf gelenkspezifische Risikofaktoren.

In der zweiten Runde wurden die 70 gelenkspezifischen Faktoren beurteilt und anschließend nach Relevanz priorisiert. In der Summe wurden 18 Faktoren positiv bewertet und daher in die dritte Runde übernommen, 52 weitere gelenkspezifische Faktoren wurden insgesamt negativ bewertet und ausgeschlossen (Tab. 2).

**Tab. 2:** Schlüsselergebnisse der drei Runden des Delphi-Verfahrens.

1. Runde	2. Runde	3. Runde
*Strukturierter Fragebogen*	*Strukturierter Fragebogen*	*Virtuelles Konsensus-Meeting*
*Input:*	*Input:*	*Input:*
Alle 120 Risikofaktoren	70 gelenkspezifische Risikofaktoren	18 gelenkspezifische Risikofaktoren
*Entscheidungskriterium:*	*Entscheidungskriterium:*	*Entscheidungskriterium:*
Bewertung −2 bis +2	Bewertung +/−	Auswahl nach Ranking
*Schlüsselergebnisse:*	*Schlüsselergebnisse:*	*Schlüsselergebnisse:*
Entscheidung Fokus auf Gelenkspezifische Risikofaktoren	18 gelenkspezifische Risikofaktoren, Ranking nach Wichtigkeit	Wichtigste 12 gelenkspezifische Risikofaktoren
24 zusätzliche Risikofaktoren aus Kommentaren		Ranking der Risikofaktoren
		Definition der Risikofaktoren (Gelenkwinkel etc.)

In der dritten Runde definierte das Panel die Kriterien präziser (z. B. Winkelangaben bei Achsfehlstellungen) und einigte sich konsensbasiert auf die 12 wichtigsten Risikofaktoren für das Auftreten von Komplikationen nach Implantation einer Knie-TEP (Tab. 3).

**Tab. 3:** Ergebnis des dreistufigen Delphi-Verfahrens, 12 gelenkspezifische Risikofaktoren für das Auftreten von Komplikationen bei der Implantation von Kniegelenksendoprothesen.

Ranking	12 gelenkspezifische Risikofaktoren	ICD-10 (wenn vorhanden)
1	Septische Voroperationen	(B94.9) nicht spezifisch kodierbar
2	Große Knochendefekte femoral oder tibial („complex primary“)	(M89.56) nicht spezifisch kodierbar
3	Einliegendes Fremdmaterial	(Z98.88) nicht spezifisch kodierbar
4	Valgusfehlstellung > 10°	(M21.06) nicht spezifisch kodierbar
5	Varusfehlstellung > 15°	(M21.16) nicht spezifisch kodierbar
6	Streckdefizit > 10°	(M24.56, M25.66) nicht spezifisch kodierbar
7	Flexion < 70°	(M24.56, M25.66) nicht spezifisch kodierbar
8	Kellgren-&-Lawrence-Score < 3°	Nicht kodierbar
9	Patella baja (Caton-Deschamps index < 0,6) [10]	(M22.8) nicht spezifisch kodierbar
10	Knöcherne Voroperation	(Z98.88) nicht spezifisch kodierbar
11	Genu recurvatum > 10°	(M21.86) nicht spezifisch kodierbar
12	Vorausgegangene Bandrekonstruktionen	(Z98.88) nicht spezifisch kodierbar

*ICD-10* International Statistical Classification of Diseases and Related Health Problems 10

## Diskussion

Diese Studie identifizierte erstmals 12 gelenkspezifische Risikofaktoren für Komplikationen nach Knieendoprothetik in Deutschland und priorisierte sie anhand eines strukturierten Delphi-Verfahrens. Damit liegt eine wissenschaftlich fundierte Grundlage vor, um bestehende Risikoadjustierungsmodelle gezielt zu erweitern und die Vergleichbarkeit von Behandlungsergebnissen zu verbessern.

Die Bedeutung präziser Risikoadjustierungsmodelle nimmt angesichts wachsender Transparenzforderungen stetig zu. Mit Einführung des Bundes-Klinik-Atlas [7] können Patientinnen und Patienten die Behandlungsqualität verschiedener Zentren direkt vergleichen. Damit diese Vergleiche fair und aussagekräftig sind, sollten Risikomodelle künftig sowohl patienten- als auch gelenkspezifische Faktoren berücksichtigen.

Derzeit berücksichtigen Risikomodelle in Deutschland überwiegend patientenbezogene Faktoren aus Routinedaten, wie Alter, Geschlecht, BMI und Komorbiditäten, darunter Diabetes mellitus, kardiovaskuläre Erkrankungen oder Nikotinkonsum [3–5, 11, 14, 27–30, 33]. Häufig wird hierzu der Elixhauser-Score [15] herangezogen, beispielsweise in den Risikoadjustierungen des WidO [34]. Solche Modelle liefern wertvolle epidemiologische und sozioökonomische Erkenntnisse, basierend auf Routinedaten (Sekundärdaten). Die Verwendung von Sekundärdaten ist jedoch für die individuelle Risikoprognose häufig zu unspezifisch [4, 16].

So wurden z. B. für die Mortalitätsbewertung nach Knie-TEP durch das IQTIG im Jahr 2024 die Faktoren Geschlecht, Alter, Gehstrecke bei Aufnahme, Verwendung von Gehhilfen, ASA-Klassifikation, präoperative Wundkontamination, periprothetische Fraktur als Indikator und Implantation einer unikondylären Schlittenprothese berücksichtigt [22]. Diese in der Sekundärbewertung berücksichtigten Faktoren scheinen eine relevante Rolle einzunehmen, repräsentieren jedoch nur teilweise die aus klinischer Perspektive entscheidenden Risikofaktoren. Während die Nutzung von prozedurspezifischen Daten (Primärdaten) aus klinischer Sicht wünschenswert wäre und den Vorteil einer hohen Validität bietet, ist sie mit erheblichem Aufwand in Bezug auf Zeit, Kosten und personelle Ressourcen verbunden. Sekundärdaten sind dagegen flächendeckend und einfach verfügbar. Jedoch weisen Sekundärdaten inhärente Limitationen wie potenzielle Ungenauigkeiten, eine eingeschränkte Verwendbarkeit zur spezifischen Forschungsfragestellung sowie eine retrospektiv schlecht beurteilbare Datenqualität auf. Diese Differenzierung zwischen Primär- und Sekundärdaten ist insbesondere bei der Qualitätssicherung von zentraler Bedeutung. Zusammenfassend erweist sich ein flächendeckender Einsatz risikoadjustierter Modelle auf Grundlage von Primärdaten als nicht realisierbar. Umso essenzieller ist daher die Identifikation weniger, jedoch hochgradig klinisch relevanter Risikofaktoren, die in Sekundärdaten abgebildet und für die Analyse nutzbar gemacht werden können.

Die American Association of Hip and Knee Surgeons (AAHKS) hat daher eine Tabelle von Risikofaktoren veröffentlicht und empfiehlt ihren Mitgliedern, diese systematisch zu dokumentieren ([2]; Tab. 4). Neben gelenkspezifischen Parametern umfasst die AAHKS-Liste auch patientenbezogene Faktoren wie Adipositas, Nikotinkonsum oder psychische Vorerkrankungen.

**Tab. 4:** Von der AAHKS vorgeschlagene Risikofaktoren zur Verbesserung der Risikoadjustierung in der Kniegelenksendoprothetik (https://www.aahks.org/practice-resources/risk-stratification/).

„Clinical risk factor“	ICD-10-Code	„Descriptor“
„Morbid obesity“ BMI > 40	E66.09	„Morbid (severe) obesity due to excess calories“
„Smoking“	Z72.0	„Tobacco use“
„Chronic anticoagulant use“	Z79.01	„Long-term (current) use of anticoagulants“
„Chronic narcotic use“	F11.20	„Opioid dependence, uncomplicated“
„Workmen’s compensation case“	Z56.9	„Unspecified problems related to employment“
„Previous intra-articular infection“	B94.9	„Sequelae of unspecified infectious and parasitic diseases“
„Angular knee deformity > 15 degrees“	M21.869	„Other acquired deformity of knee“
„Previous ORIF knee“	M17.31	„Unilateral post-traumatic osteoarthritis, right knee“
	M17.32	„Unilateral post-traumatic osteoarthritis, left knee“
„Depression/psychiatric disease“	F48.9	„Nonpsychotic mental disorder“

*BMI *Body-Mass-Index,* ICD-10* International Statistical Classification of Diseases and Related Health Problems 10, *ORIF „*open reduction and internal fixation“

Internationale Erfahrungen verdeutlichen die möglichen Konsequenzen einer unzureichenden Risikoadjustierung: In den USA beeinflussen Programme wie „Quality Adjusted Payment“ und „Bundle Payment“ die Vergütung von Kliniken direkt. Dies führte teilweise dazu, dass komplexere Hochrisikopatienten gemieden wurden („cherry picking“ und „lemon dropping“) [1, 6, 12, 17, 20, 26, 36], was bereits ethische Diskussionen auslöste [21]. Sollten vergleichbare Vergütungsstrukturen in Deutschland eingeführt werden, ist eine umfassende und praxisgerechte Risikoadjustierung zwingend erforderlich, um Kliniken mit hohem Anteil komplexer Fälle – insbesondere Universitätskliniken und andere Maximalversorgungshäuser – nicht zu benachteiligen.

In Deutschland bestehen zudem erhebliche Defizite bei den Verfahren zur Überprüfung der Indikationsqualität in der Knieendoprothetik. Die Evaluation der datengestützten Qualitätssicherung im Rahmen des G‑BA-Eckpunktepapiers vom 22. April 2022 kam für das Verfahren QS KEP zu dem Ergebnis, dass die Qualitätsindikatoren zur Indikationsstellung nicht weiterempfohlen werden können. Die aktuelle Form der Datenerfassung sei nicht ausreichend evidenzbasiert, und die Messeigenschaften zur Bewertung einer bedarfsgerechten Indikation erwiesen sich als unzureichend. (Abschlussbericht „Weiterentwicklung der datengestützten QS: Verfahren KEP“ vom 19. Juli 2023). Für das Jahr 2025 wurde die Erhebung für QS-Daten in der Knieendoprothetik vom IQTIG daher ausgesetzt [23].

Eine spezifischere Risikoadjustierung für die Knieendoprothetik ist aktuell noch nicht möglich. Einerseits sind viele gelenkspezifische Risikofaktoren nicht in ICD-Codes abbildbar und fehlen daher in den Routinedaten. Andererseits werden die bestehenden Kodiermöglichkeiten hinsichtlich der Dokumentation von Risikofaktoren nicht voll ausgeschöpft und sind in der Regel auf die Kodierung vergütungsrelevanter Codes beschränkt. Hinzu kommt, dass eine detaillierte Erfassung zusätzlicher Risikofaktoren in den Kliniken erheblichen Mehraufwand bedeutet. Daher muss sorgfältig abgewogen werden, in welchem Umfang eine Erweiterung der Dokumentation zur Verbesserung der Risikoadjustierung erforderlich und realisierbar ist [32].

### Praktische Implikationen

Die Ergebnisse dieser Studie bilden eine Grundlage für die Weiterentwicklung der Qualitätssicherung in der Knieendoprothetik. Kurzfristig sollten die identifizierten Risikofaktoren in klinischen Kohorten validiert und mit Routinedaten verknüpft werden. Mittelfristig ist eine präzisere Kodierung erforderlich, um gelenkspezifische Faktoren zuverlässig abzubilden. Langfristig könnten Risikoadjustierungsmodelle so erweitert werden, dass sie patienten- und gelenkspezifische Faktoren integrieren und damit sowohl eine gezieltere Optimierung des perioperativen Managements als auch eine präzisiere Bewertung von Behandlungsergebnissen ermöglichen.

### Limitationen

Die Aussagekraft des Delphi-Verfahrens hängt maßgeblich von der Zusammensetzung des repräsentativen Panels ab [9, 31, 35]. Ein Selektionsbias kann nicht ausgeschlossen werden, da die Teilnahme vermutlich auf besonders motivierte Experten beschränkt war [24]. Zudem müssen die identifizierten Risikofaktoren noch in klinischen Kohorten validiert werden. Schließlich kann eine Risikoadjustierung nur funktionieren, wenn alle Kliniken konsequent dokumentieren – derzeit sind jedoch nicht alle Risikofaktoren durch ICD-Codes abbildbar.

## Schlussfolgerung

Dieses strukturierte Delphi-Konsensusverfahren identifizierte erstmals 12 gelenkspezifische Risikofaktoren in der Knieendoprothetik. Sie stellen eine relevante Ergänzung bestehender Risikoadjustierungsmodelle dar und könnten die Vergleichbarkeit von Behandlungsergebnissen verbessern. Zukünftige Studien sollten ihre Validität prüfen und die Integration in Routinedatenregister und Qualitätssicherungsinstrumente untersuchen, um langfristig eine präzisere und fairere Qualitätsbewertung zu ermöglichen.

## Data Availability

Die erhobenen Datensätze können auf begründete Anfrage in anonymisierter Form beim korrespondierenden Autor angefordert werden. Die Daten befinden sich auf einem Datenspeicher am Klinikum rechts der Isar der Technischen Universität München.

## Abkürzungen

AAHKS: American Association of Hip and Knee Surgeons
AWMF: Arbeitsgemeinschaft der Wissenschaftlichen Medizinischen Fachgesellschaften e. V.
DGOOC: Deutsche Gesellschaft für Orthopädie und Orthopädische Chirurgie
EPRD: Endoprothesenregister Deutschland
IGTIQ: Institut für Qualitätssicherung und Transparenz im Gesundheitswesen
RAND: Research and development corporation (Think Tank in den USA)
TEP: Totalendoprothese
WidO: Wissenschaftliches Institut der AOK
